# Dicer-Like Proteins Regulate Sexual Development via the Biogenesis of Perithecium-Specific MicroRNAs in a Plant Pathogenic Fungus *Fusarium graminearum*

**DOI:** 10.3389/fmicb.2018.00818

**Published:** 2018-04-26

**Authors:** Wenping Zeng, Jie Wang, Ying Wang, Jing Lin, Yanping Fu, Jiatao Xie, Daohong Jiang, Tao Chen, Huiquan Liu, Jiasen Cheng

**Affiliations:** ^1^State Key Laboratory of Agricultural Microbiology, Huazhong Agricultural University, Wuhan, China; ^2^The Provincial Key Lab of Plant Pathology of Hubei Province, College of Plant Science and Technology, Huazhong Agricultural University, Wuhan, China; ^3^State Key Laboratory of Crop Stress Biology for Arid Areas, College of Plant Protection, Northwest A&F University, Yangling, China

**Keywords:** microRNAs, sexual development, perithecium-specific milRNAs, dicer-like protein, *Fusarium graminearum*

## Abstract

Ascospores act as the primary inoculum of *Fusarium graminearum*, which causes the destructive disease Fusarium head blight (FHB), or scab. MicroRNAs (miRNAs) have been reported in the *F. graminearum* vegetative stage, and *Fgdcl2* is involved in microRNA-like RNA (milRNA) biogenesis but has no major impact on vegetative growth, abiotic stress or pathogenesis. In the present study, we found that ascospore discharge was decreased in the *Fgdcl1* deletion mutant, and completely blocked in the double-deletion mutant of *Fgdcl1* and *Fgdcl2*. Besides, more immature asci were observed in the double-deletion mutant. Interestingly, the up-regulated differentially expressed genes (DEGs) common to *ΔFgdcl1* and *ΔFgdcl1/2* were related to ion transmembrane transporter and membrane components. The combination of small RNA and transcriptome sequencing with bioinformatics analysis predicted 143 novel milRNAs in wild-type perithecia, and 138 of these milRNAs partly or absolutely depended on *Fgdcl1*, while only 5 novel milRNAs were still obtained in the *Fgdcl1* and *Fgdcl2* double-deletion mutant. Furthermore, 117 potential target genes were predicted. Overall, *Fgdcl1* and *Fgdcl2* genes were partly functionally redundant in ascospore discharge and perithecium-specific milRNA generation in *F. graminearum*, and these perithecium-specific milRNAs play potential roles in sexual development.

## Introduction

*Fusarium graminearum* (teleomorph *Gibberella zeae)* is one of the major pathogens that cause Fusarium head blight (FHB) in barley, maize and other grains (Bai and Shaner, 2004; Goswami and Kistler, 2004). Ascospores act as the primary inoculum of plant infections by releasing from perithecia and then falling on flowering spikelets (Trail et al., 2002). Therefore, sexual reproduction and ascospore discharge play a critical role in head blight epidemiology. In sexual reproduction, in addition to MAT genes, ion transport proteins (such as potassium, chloride, and calcium icon channels) and certain transcription factors are associated with perithecium formation and discharge mechanism (Hallen et al., 2007; Kim et al., 2015). Interestingly, *Fgago2*, which is related to the RNA interference pathway, is associated with meiosis and subsequent developmental pathways in *F. graminearum* (Kim et al., 2015). In *Neurospora crassa, dcl-1*, an RNA silencing regulator, also effected sexual development at early stage (Alexander et al., 2008). The RNAi component mutants of these ascomycetes affect sexual development, suggesting that certain endogenous short RNAs (esRNAs) play regulatory roles during sexual development.

Recently, several types of esRNAs have been described in fungi, such as exonic-small interfering RNAs (ex-siRNAs) in *Mucor circinelloides* and *F. graminearum*, tRNA-derived fragments (tRFs) in *Magnaporthe oryza*e, heterochromatin-derived short-interfering RNAs (siRNAs) in *Schizosaccharomyces pombe*, QDE-2-interacting sRNAs (qiRNAs), and microRNA-like RNAs (milRNAs) in *N. crassa* (Volpe et al., 2002; Lee et al., 2009, 2010; Nicolas et al., 2010; Nunes et al., 2011; Son et al., 2017). MicroRNAs (miRNAs), the most common example of esRNAs in plants and animals, also have been detected in several fungal organisms, such as *Sclerotinia sclerotiorum, Cryptococcus neoformans, Trichoderma reesei, Fusarium oxysporum, F. graminearum, Penicillium marneffei*, and *Penicillium chrysogenum* (Jiang et al., 2012; Zhou et al., 2012; Kang et al., 2013; Lau et al., 2013; Chen et al., 2014, 2015; Dahlmann and Kuck, 2015). miRNAs are produced by a canonical Dicer-dependent biogenesis pathway and control gene expression at the post-transcriptional level in plants and animals (Ambros et al., 2003). In *N. crassa*, Dicer, Argonaute, RNA-dependent RNA polymerase (RdRp), the RNase III domain-containing protein MRPL3, and RNA exosomes play important roles in milRNA biogenesis pathways (Lee et al., 2010; Xue et al., 2012). Animal miRNAs recognize their targets by the miRNA seed region; nevertheless, plant miRNAs nearly perfectly matched to their targets (Rhoades et al., 2002; Lewis et al., 2003). Although miRNAs have been found in various fungi, there is little information on their function and target recognition.

*Fusarium graminearum*, similar to major ascomycete fungi, contains two Dicer-like and two Argonaute-like protein coding genes (Nakayashiki, 2005). In the *F. graminearum* asexual stage, the RNAi components are not involved in vegetative development or pathogenesis (Chen et al., 2015). In stark contrast, *Fgdcl1* and *Fgago2* affect ascospore formation during the sexual phase (Son et al., 2017). Indeed, we speculated that certain sex-induced milRNAs exist in perithecium and play regulatory functions in sexual reproduction. To gain insights into these milRNAs, we predicted the wild-type strain PH-1 milRNAs before (0 dps) and after (7 dps) self-crossing and compared the perithecium-specific milRNAs of the *Fgdcl-1* (*FGRRES_09025*) and *Fgdcl1/2* (the gene id of *Fgdcl2* is *FGRRES_04408*) deletion mutants with those in PH-1 via deep sequencing and bioinformatics analysis. To explore the function of milRNA, we also predicted and analyzed milRNA target genes.

## Materials and Methods

### Fungal Strains and Culture Conditions

The *F. graminearum* strain PH-1 (NRRL 31084) (Trail and Common, 2000) was used as the wild-type strain in the present study, and mutant strains were derived from the wild-type strain. All strains in this study were cultured on potato dextrose agar (PDA) plates at 25°C and stored in PDA slants at 4°C.

To characterize the biological properties of asexual stage, colony morphology, hyphal extensions, conidial production, and conidial germination were examined. The hyphal extension rate (mm day^-1^) was measured on PDA, complete medium (CM) and minimal medium (MM) (Correll et al., 1987) at 25°C between 1 and 3 days using three replicate plates per strain. Colony morphology was observed on PDA media at 25°C for 3 days. To assay the sensibility of various stresses, one mycelial plug was centrally inoculated in PDA medium containing 1 M glucose, 1 M sorbitol, 1 M NaCl, 1 M KCl, 0.5 M CaCl_2_, 0.02% SDS, 0.05% H_2_O_2_, or 0.2 mg/ml Congo red. For conidiation assays, one mycelial plug was inoculated into 20 ml of carboxymethyl cellulose (CMC) (1 g NH_4_NO_3_, 1 g KH_2_PO_3_, 0.5 g MgSO_4_.7H_2_O, 1 g yeast extract, 15 g CMC, and 1 L of water) liquid medium at 25°C for 5 days in a shaker (200 rpm), and the number of conidia was assessed using a hemocytometer (Cappellini and Peterson, 1965). For conidial germination, conidia were harvested and then inoculated into YEPD (0.3% yeast extract, 1% peptone, and 2% dextrose) liquid medium at 25°C in a shaker (200 rpm), and then conidial germination was observed and counted after 4 and 6 h of incubation.

### Sexual Development and Ascospore Discharge

With certain modifications, the sexual development and ascospore discharge assays *in F. graminearum* were performed as previously described (Leslie et al., 2006; Cavinder et al., 2012). Briefly, one mycelial plug was inoculated onto carrot plats at 25°C for 7 days and then removed with the back of a surgical blade and added to 600 μl of 2.5% Tween 60 solution to induce sexual development. The sample was continuously incubated under near-UV light (wavelength, 365 nm; beauty bright Lighting Electrical Appliance Co., Ltd., Zhongshan, China) at 23°C. After induction for 7 days induction, perithecia and asci were observed under dissecting and light microscopes, respectively. The glycogen staining of asci was performed as previously reported (da R Campos and Costa, 2010). Ascospore discharge was detected in a coverslip and place in a closed box. A semicircular agar block (10 mm in diameter) covered with mature perithecia was placed on a coverslip for 24 h, and the images were captured on camera (Trail et al., 2005; Min et al., 2010; Cavinder et al., 2012).

### Plant Infection Assays

Infection assays on flowering wheat heads were performed as previously described (Hou et al., 2002). To this end, 10 μl of 1 × 10^5^ conidia/ml conidia suspension, collect from 5-day-old CMC cultures, was inoculated onto the fifth flowering spikelet of Zhengmai 9023. The symptomatic spikelets were examined and images were captured at 20 days after inoculation. Corn silk infection assay was performed according to Seong et al. (2005). Corn silks were inoculated with mycelia plugs (diameter = 6 mm) and counted at 6 dpi.

### RNA Extraction and Quantitative Real Time (qRT)-PCR

In the asexual stage, the RNA samples were isolated from 24-h YEPD cultures (mycelium) and 24-h CMC cultures (sporulation). During sexual development, the RNA samples were collected from 7-day carrot plat hyphae (0 day post self-crossing, 0 dps) and 3-, 5-, 7-day perithecia (3, 5, and 7 days post self-crossing). Total RNA was isolated with the RNAiso Plus regent (Takara, Shiga, Japan) according to the manufacturer’s protocols. Potential DNA contamination was removed, and first-strand cDNA was synthesized by the EasyScript One-step gDNA Removal and cDNA Synthesis SuperMix (Transgen Biotech, Beijing, China) according to the manufacturer’s instructions.

The gene transcript levels were assessed by quantitative real-time reverse transcriptase PCR (qRT-PCR). qRT-PCR was performed on a CFX96 Real-Time PCR Detection System (Bio-Rad, Hercules, CA, United States) with the iTaq Universal SYBR Green Supermix (Bio-Rad, Hercules, CA, United States USA). The *F. graminearum actin* gene was used as the internal control. The gene expression was calculated with the 2^-ΔΔCt^ method, and the mean and standard deviation were calculated from three biological replicates (Livak and Schmittgen, 2001). The gene-specific primer pairs for qRT-PCR are listed in **Supplementary Table S1**.

### DNA Manipulation and Fungal Transformations

The strategy for gene deletion is illustrated in **Supplementary Figure S1** according to a split-marker system (see Supplementary Materials) (Catlett et al., 2003). PCR products for targeted gene deletion were constructed by a slightly modified double-joint (DJ) PCR. First, the upstream and the downstream fragments of *Fgdcl2* were amplified from genomic DNA using the primer pair dcl2-1F1/1R and dcl2-2F/2R1, respectively (**Supplementary Table S1**). Next, the hygromycin resistance gene (HPH) was amplified from PUCH18 using the primer pair HYG-F/HYG-R (**Supplementary Table S1**; Yang et al., 2018). The three amplicons (the upstream fragment, the downstream fragment, and HPH) were fused after a second round of DJ-PCR. Finally, two DNA fragments were amplified from the second-round product with the primer pairs dc12-1F1/HY-R and YG-F/dcl2-2R1 (**Supplementary Table S1**). These two DNA fragments were subsequently transformed into protoplasts of wild-type strain PH-1 according to a previously published protocol (Kim et al., 2006; Han et al., 2007). For *Fgdcl1* and *Fgdcl*2 double deletion, the geneticin resistance cassette (gen) was used as a selectable marker. Transformations were selected by using the PDA plates amended with 225 μg ml^-1^ hygromycin B (Sigma-Aldrich) or 300 μg ml^-1^ geneticin (Sigma-Aldrich). Transformants were purified by single conidium isolation and stored in 20% glycerol at -80°C.

For Southern blotting, the genomic DNAs of PH-1 and mutants were extracted according to the Fusarium laboratory manual and then digested with XbaI (Leslie et al., 2006). Southern blotting was performed using the Amersham AlkPhos Direct Labeling and Detection System (GE Healthcare, Little Chalfont, United Kingdom). The probe primers are listed in **Supplementary Table S1** and the probes were labeled with alkaline phosphatase.

### RNA Isolation and Library Construction

Mature perithecia (7 days post self-crossing) of the wild-type strain PH-1 and mutants (*ΔFgdcl1 and ΔFgdcl1/2*) were isolated to construct sexual stage small RNA and cDNA libraries. The mycelia of PH-1 were inoculated onto carrot plate for 7 days and collected as 0 day post self-crossing (0 dps) sample. Total RNA was extracted using the RNAiso Plus regent (Takara, Shiga, Japan) according to the manufacturer’s instructions and then treated with RNase-free DNase I. RNA quality was measured using a NanoDrop 2000 spectrophotometer (Thermo Scientific, United States) and Agilent 2100 Bioanalyzer (Agilent, United States). Small RNAs (15∼30 nt) were extracted from total RNA on a 15% denaturing polyacrylamide gel and ligated to specific 5′ adaptor and 3′ adaptor samples. After reverse transcription, the cDNA libraries were sequenced on an Illumina HiSeq 2000 platform (BGI, Shenzhen, China). For each strain, one small RNA library and one transcriptome library were constructed. For each strain, two biological replicates were used. The RNA-seq and sRNA-seq data were deposited in the NCBI Sequence Read Archive database with accession codes SRP132245 and SRP131559, respectively.

### Data Analysis of Small RNA and Transcriptome

The small RNA analysis and microRNA prediction were performed as previously reported (Zhou et al., 2012). After the removal of poor-quality reads and adaptor sequences, clean reads were obtained with the FASTX toolkit. Small sequence reads were completely mapped to the genome of PH-1 from the Ensembl Fungi database using bowtie (King et al., 2015). After removal of known non-coding RNAs (rRNA, tRNA, snRNA, and snoRNA) by BLAST or bowtie, the unannotated small RNAs were used for novel miRNA prediction by using MIREAP (Li et al., 2012). The raw abundance of milRNAs was normalized according to transcripts per million (TPM) normalization, where the n_base is 1,000,000 (Jeong et al., 2010; Belli Kullan et al., 2015). PsRobot (Wu et al., 2012) and TargetFinder (Lavorgna et al., 1999) were used for the prediction of miRNA according to plant-like target interactions, and based on the methods described by Allen with slightly modified: (1) less than four mismatches in total and no more than two adjacent mismatches; (2) In positions 1–12, no more than 2.5 mismatches; (3) after 17 position, one gap or bulge was permitted.

The transcriptome analysis in *F. graminearum* was performed as previously reported (Cao et al., 2016). The RNA-seq reads were mapped to the genome of *F. graminearum* strain PH-1 with Tophat2, and then the transcript levels were calculated by FeatureCounts (Kim et al., 2013; Liao et al., 2014). Differential expression analysis of genes was performed with the edgeRun package (Dimont et al., 2015). Genes with a false discovery rate (FDR) below 0.05 were considered differentially expressed. Gene ontology (GO) functions were enriched according to Young et al. (2010) reported, the *p*-value was calculated and subjected to Benjamini-Hochberg correction, with adjusted *p*-values ≤ 0.05 as a threshold. In addition, we summarized and visualized the GO terms employing REVIGO (Bauer et al., 2008; Supek et al., 2011).

## Results

### *Fgdcl1* Partly Cooperates With *Fgdcl2* in Ascospore Discharge

**FIGURE 1:**
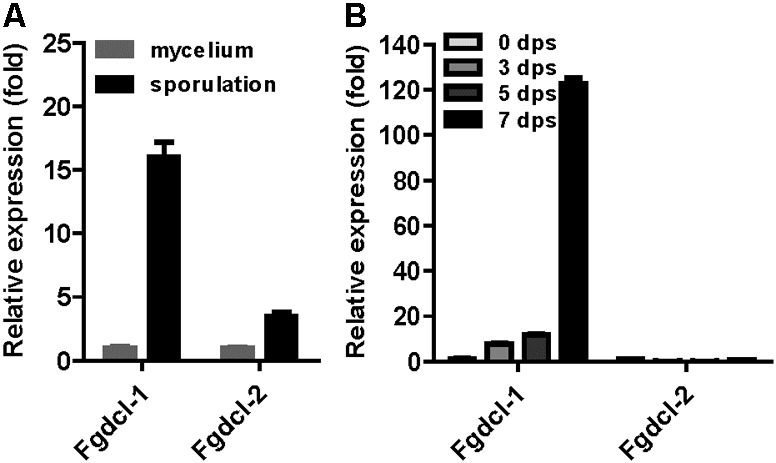
The expression patterns of *Fgdcl* gene in asexual and sexual stages. **(A)** The expression patterns of *Fgdcl* gene in asexual stages. In asexual stage, cultures were collected from YEPD and carboxymethyl cellulose (CMC) liquid medium after 24 h inoculation as mycelium and sporulation samples, respectively. The expression of *Fgdcl* genes in mycelium was set as the control. **(B)** The expression patterns of *Fgdcl* gene in sexual stage. The perithecium was collected from carrot plate after 3, 5, and 7 days of self-crossing. The mycelium collected from carrot plate for 7-day inoculation was considered as the 0 day post self-crossing (0 dps) sample. The expression of *Fgdcl* genes at 0 dps was set as the control. The relative mRNA expression of the *Fgdcl* genes was determined by quantitative RT-PCR. Bars indicate standard the deviation from three repeated experiments.

**FIGURE 2:**
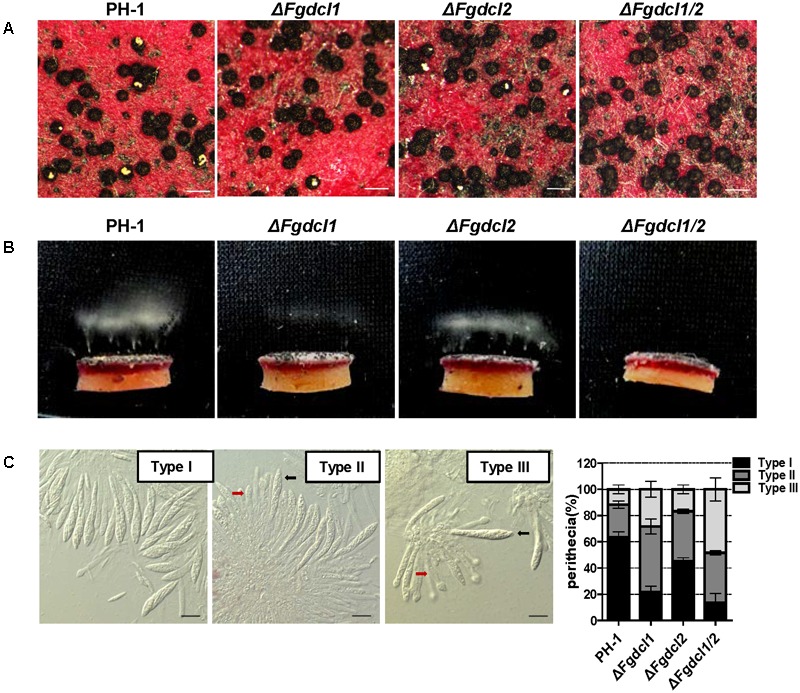
Comparison of the sexual development among the wild-type strain PH-1, *ΔFgdcl1, ΔFgdcl2* and *ΔFgdcl1/2.* **(A)** Comparison of the cirrhi of wild-type strain PH-1, *ΔFgdcl1, ΔFgdcl2* and *ΔFgdcl1/2*. Each strain was inoculated on carrot medium, and the images were captured at 12 days post self-crossing (dps). The yellow cirrhi was the overflowed ascospores from the ostiole of perithecia. Scale bar = 200 μm. **(B)** Ascospore discharge was assayed with 7-day-old perithecia. The white cloud is the accumulation of discharged ascospores. The images were captured after incubation for 20 h. **(C)** Morphology and statistical analysis of asci rosettes of 7-day-old perithecia. The morphology of type I asci rosettes was selected from the wild-type strain PH-1; type II and type III were selected from *ΔFgdcl1/2*. The black and red arrows indicate the normal and defective asci, respectively. Bar = 20 μm. The statistical analysis of ascus morphology was conducted after 7 days post self-crossing. A total of 50 asci rosettes were observed and classified in each strain with light microscopy. Line bars indicate standard errors from three repeated experiments.

A previous study has shown that the RNAi components not involved in vegetative development and pathogenesis in *F. graminearum* (Chen et al., 2015). Therefore, we analyzed the expression patterns of *Fgdcl* genes in asexual and sexual stage. The results showed that the expression level of *Fgdcl1* in sporulation was higher than those in mycelium (**Figure 1A**), similar to previous research (Chen et al., 2015). Meanwhile, compared to 0 dps (0 day post self-crossing), the expression level of *Fgdcl1* was markedly increased after sexual induction for 3, 5, and 7 days, especially at 7 dps (**Figure 1B**). To explore whether *Fgdcl* plays a physiological role in sexual development, we constructed the gene deletion mutants *ΔFgdcl1*, Δ*Fgdcl2*, and *ΔFgdcl1/2*. These mutants were confirmed by PCR amplification and Southern blotting (**Supplementary Figure S1**). In the asexual stage, no significant changes in hyphal growth, conidiation, conidia morphology, or virulence and under various environmental stress conditions were observed (**Supplementary Figures S2, S3**). During sexual development, normal-shaped perithecia were obtained from these mutants at 7 days after fertilization. In contrast to the perithecia in PH-1 and Δ*Fgdcl2*, few cirrhi were produced in *ΔFgdcl1*, and *ΔFgdcl1/2* (**Figure 2A**). The discharged ascospores were decreased in *ΔFgdcl1* and almost abolished in *ΔFgdcl1/2*, compared with those in PH-1 (**Figure 2B**). The dissection of perithecia showed that less than 20% perithecia had normal asci (type I) containing eight spindle-shaped ascospores in *ΔFgdcl1* and *ΔFgdcl1/2*, while 63% and 45% type I asci were observed in PH-1 and Δ*Fgdcl2*, respectively (**Figure 2C**). In *ΔFgdcl1* and *ΔFgdcl1/2*, most of the asci were defective, such as abnormal asci with fewer ascospores (type II) and smaller asci (type III) (**Figure 2C**). Indeed, the ascospore discharges was partly decreased in *ΔFgdcl1* and arrested in *ΔFgdcl1/2* due to the defective asci. Moreover, similar results were obtained from other deletion mutants (**Supplementary Figure S4**). Taken together, these findings reveal that *Fgdcl1* plays a more important role in sexual reproduction than in the asexual stage and that *Fgdcl1* partly cooperates with *Fgdcl2* in ascospore discharge.

### The Common Up-Regulated DEGs in *ΔFgdcl1* and *ΔFgdcl1/2* Involved in Ascospore Discharge

**FIGURE 3:**
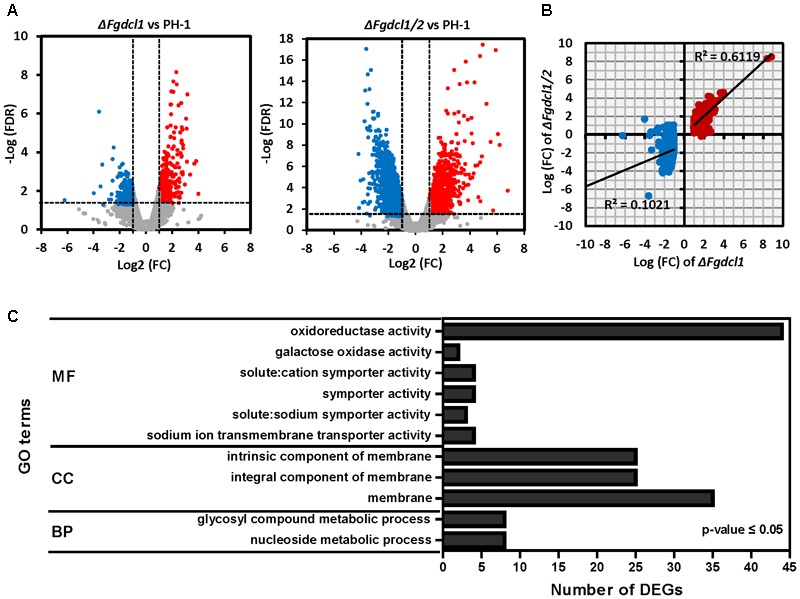
Transcriptomics analysis of the wild-type strain PH-1, *ΔFgdcl1*, and *ΔFgdcl1/2* in sexual stage. **(A)** Volcano plots of up/down-regulated differentially expressed genes (DEGs) and non-differentially expressed genes between *ΔFgdcl1* and PH-1, and between *ΔFgdcl1/2* and PH-1. The red and blue dots indicate the up-regulated and down-regulated DEGs, respectively. The gray dots indicated non-differentially expression genes. Genes with an false discovery rate (FDR) below 0.05 and fold changes greater than two were considered as DEG. **(B)** Correlation analyses of DEGs of *ΔFgdcl1* and *ΔFgdcl1/2.* The red and blue dots indicate the up-regulated and down-regulated DEGs in *ΔFgdcl1*, respectively. **(C)** The Gene ontology (GO) enrichment of common up-regulated DEGs between *ΔFgdcl1* and *ΔFgdcl1/2*. The shown GO terms act as the significant enrichment terms, with *p*-values less than 0.05, adjusted by Benjamini-Hochberg correction. BP, biological process; CC, cellular component; MF, molecular function.

To identify the genes regulated by *Fgdcl1* and investigate the molecular mechanisms underlying ascospore discharge, the transcriptomes at 7 dps perithecia of the wild-type strain PH-1, as well as *ΔFgdcl1* and *ΔFgdcl1/2* were obtained using the Illumina HiSeq^TM^ 2000 platform (BGI, Shenzhen, China), and the reads were mapped to *F. graminearum* genome (**Supplementary Table S2**). Genes with a FDR of less than 0.05 and a fold-change greater than 2 compared with the wild-type strain PH-1 were considered differentially expressed genes (DEGs). In total, 846 DEGs were identified in *ΔFgdcl1*, including 376 down-regulated and 470 up-regulated genes (**Figure 3A** and **Supplementary Table S3**). More DEGs (2372 genes) were obtained in *ΔFgdcl1/2*, including 1170 down-regulated and 1202 up-regulated genes (**Figure 3A** and **Supplementary Table S3**). Additionally, the common up-regulated DEGs in *ΔFgdcl1* and *ΔFgdcl1/2* showed a mild positive correlation (*R*^2^= 0.6119), but this was not the case in common down-regulated DEGs, suggesting that the common up-regulated DEGs between *ΔFgdcl1* and *ΔFgdcl1/2* may share certain pathways related to ascospore discharge (**Figure 3B**). The GO annotation of 351 common up-regulated DEGs showed that 11 GO terms were enriched (adjusted *p*-value less than 0.05), such as “nucleoside metabolic” and “glycosyl compound metabolic process” in biological process; “membrane” in cellular component; and “sodium ion transmembrane transporter activity,” “symporter activity,” “galactose oxidase activity,” and “oxidoreductase activity” in molecular function (**Figure 3C**). Moreover, the network of GO terms indicated that the up-regulated DEGs were involved in transporter activity, nucleoside metabolism, transmembrane transport and membrane components (**Supplementary Figure S5**). Furthermore, certain enriched GO terms provided useful information for sexual development and ascospore discharge, including galactose oxidase activity, sodium ion trans membrane transporter activity, and solute: cation symporter activity, which may relate to the buildup of turgor pressure, an important process for ascospore discharge (Trail et al., 2002). The expression patterns of discharge-related terms gene were verified by qRT-PCR, and the results were consistent with the sequencing results (**Supplementary Figure S6A**). In addition, no significant terms were enriched in the down-regulated DEGs. Taken together, these results suggested that the common up-regulated DEGs of *ΔFgdcl1* and *ΔFgdcl1/2* may have a relationship with defective asci and ascospore discharge.

### Prediction of *F. graminearum* Perithecium-Specific milRNAs

**FIGURE 4:**
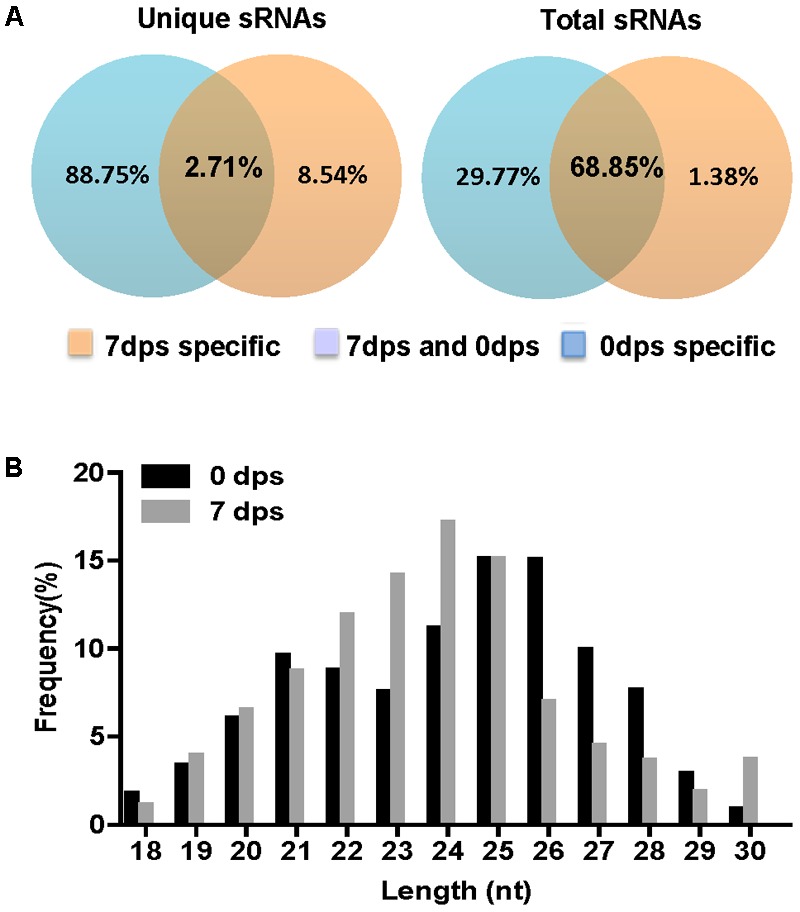
Comparison of small RNA reads among 0 day post self-crossing (dps) and 7 dps. **(A)** Venn diagram showing the public and special sRNAs between 0 dps and 7 dps. **(B)** Length distribution of the small RNAs from 0 dps and 7 dps of wild-type strain PH-1.

In the present study and previous studies, *Fgdcl1* was induced and found to play a crucial role during sexual development (**Figure 1**; Son et al., 2017). *Fgdcl2* and *Fgago1* were involved in the RNAi pathway, and the accumulation of siRNA was reduced in the *Fgdcl2* deletion mutant in the asexual stage (Chen et al., 2015). To explore whether *Fgdcl1* is involved in perithecium-specific sRNAs biogenesis, the sRNA libraries of PH-1, *ΔFgdcl1* and *ΔFgdcl1/2* were constructed and sequenced (**Supplementary Table S4**). By comparing sRNA sequences between 0 and 7 dps, 2.71% unique sRNAs were common, and 88.75% unique sRNAs were detected only in 7 dps, it means most of the sRNAs classes were obtain only from perithecia (**Figure 4A**). These results indicated that the sRNAs at 7 dps markedly differed from those at 0 dps, and most of these molecules were perithecium-specific. Analyses of the length distribution of these sRNAs revealed that these sRNAs ranged from 18–30 nt, as previously reported in fungi. Additionally, the number of 22–24 nt sRNAs at 7 dps was higher than that at 0 dps and peaked at 24 nt (**Figure 4B**).

**FIGURE 5:**
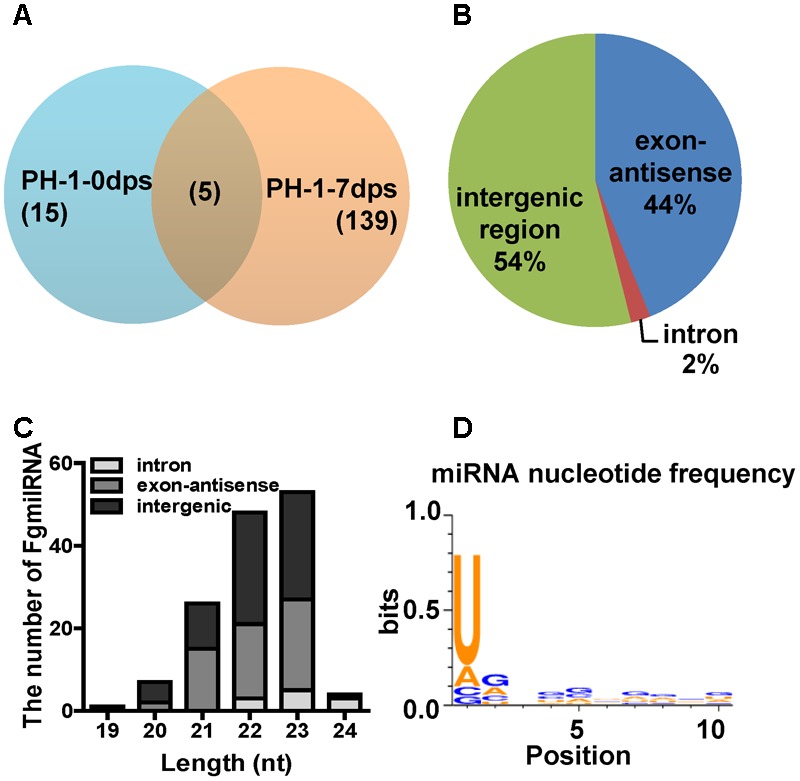
Characterization of microRNA-like RNAs (milRNAs) from *Fusarium graminearum* wild-type strain PH-1 sexual phase. **(A)** Venn diagram showing the summary of milRNAs between 0 dps and 7 dps of wild-type strain PH-1. **(B)** Distribution of pri-milRNA loci of perithecium-specific milRNAs in wild-type strain. **(C)** The length distribution of unique milRNAs and their locations. **(D)** The nucleotide frequency at 1-10 nt of unique perithecium-specific milRNAs in wild-type strain PH-1.

To predict novel miRNAs in *F. graminearum*, the small RNA sequences were mapped and annotated to the *F. graminearum* genome (**Supplementary Tables S4, S5**). Then, we predicted the micro-like RNAs from exon antisense, intron and unannotated RNAs using MIREAP (Li et al., 2012). The data showed that only 20 novel milRNAs were identified at 0 dps, while 143 novel milRNAs were generated from the mature perithecia of PH-1 (7 dps), and 139 milRNAs were detected only at 7 dps (**Figure 5A** and **Supplementary Table S6**), indicating that more milRNAs were induced during sexual reproduction. Among these perithecium-specific milRNAs, 75 (54%) were located in intergenic regions, 61 (44%) were located in antisense exon regions, and 3 (2%) were located in introns (**Figure 5B** and **Supplementary Table S6**). Compared to asexual stage milRNAs, the percentage of exon antisense loci was higher (∼two fold) in the sexual stage milRNAs (Chen et al., 2015). In addition, the unique perithecium-specific milRNAs ranged from 19 to 24 nt and were enriched in 22 and 23 nt molecules (**Figure 5C**). Moreover, a majority of 22–23 nt FgmilRNAs were located on gene antisense and intergenic regions (**Figure 5C**). Similar to most milRNAs in fungi, 73.61% of perithecium-specific milRNAs start with uracil (**Figure 5D**).

**FIGURE 6:**
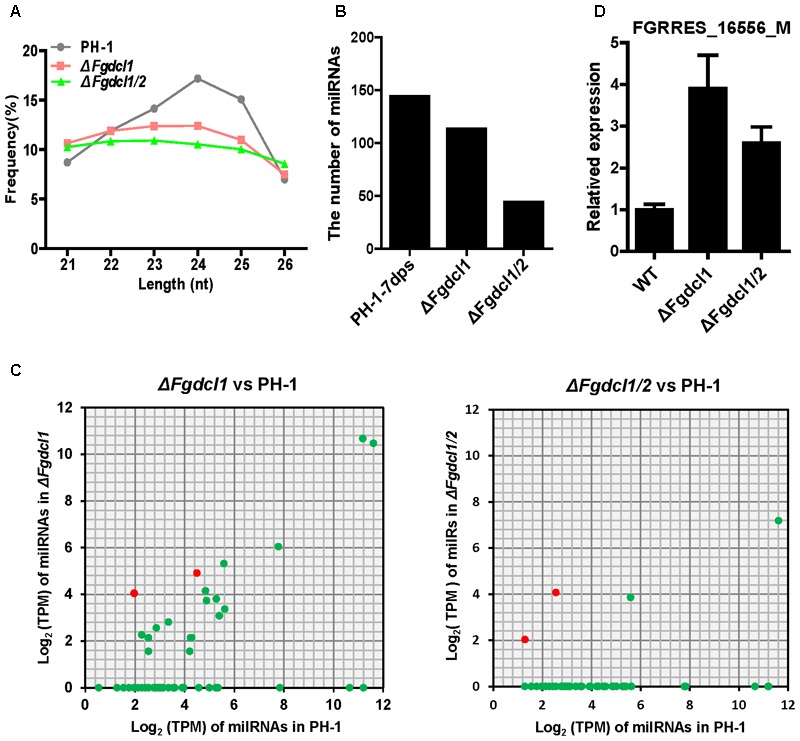
The expression patterns of milRNAs and their targets in the wild-type strain PH-1, *ΔFgdcl1* and *ΔFgdcl1/2*. **(A)** Length distribution of 21–26 nt small RNAs from 7-day-old perithecia of the wild-type strain PH-1, *ΔFgdcl1* and *ΔFgdcl1/2*. **(B)** The number of unique milRNA in the wild-type strain PH-1, *ΔFgdcl1* and *ΔFgdcl1/2*. **(C)** Comparison of the expression patterns of perithecium-specific milRNAs candidates between *ΔFgdcl1* and the wild-type PH-1, and between *ΔFgdcl1/2* and the wild-type PH-1. The expression level of milRNAs was normalized by TPM normalized formula. The milRNAs with transcripts per million (TPM) values higher than one are represented. **(D)** Relative expression of *FgmilR-7dps-61* putative target gene in the wild-type strain PH-1, *ΔFgdcl1* and *ΔFgdcl1/2*. The total RNAs were isolated from the 7-day-old perithecia of PH-1, *ΔFgdcl1* and *ΔFgdcl1/2*. The expression levels of these target genes in PH-1 served as control. Line bars indicate standard errors from three repeated experiments.

To examine whether Dicer processes these perithecium-specific milRNAs, we constructed and sequenced the sRNA libraries of *ΔFgdcl1* and *ΔFgdcl1/2*. A comparison of the sRNA libraries of PH-1, *ΔFgdcl1* and *ΔFgdcl1/2* revealed that the proportions of 23–25 nt reads were decreased in *ΔFgdcl1* and *ΔFgdcl1/2*, especially in 24 nt reads (**Figure 6A**). This finding implied that *Fgdcl1* and *Fgdcl2* may process this class of esRNAs. The prediction of milRNA showed that 113 and 44 milRNAs were obtained in *ΔFgdcl1* and *ΔFgdcl1/2*, respectively (**Figure 6B**). Nevertheless, only 18 and 4 perithecium-specific milRNAs of PH-1 could also be induced in *ΔFgdcl1* and *ΔFgdcl1/2*, respectively, and the expressions of these 18 milRNAs was lower than those in the wild-type strain (**Figure 6C** and **Supplementary Table S6**). These results indicate that most of these milRNAs may be processed by two Dicer proteins. However, *Fgdcl1-*dependent or two *Fgdcl-*dependent milRNAs showed low expression (**Figure 6C** and **Supplementary Table S6**). Furthermore, certain novel milRNAs were identified from *ΔFgdcl1* or *ΔFgdcl1/2* but not from the wild type strain PH-1.

### Role of Perithecium-Specific milRNA in the Regulation of Sexual Development of *F. graminearum*

The discharge of ascospores was reduced in *ΔFgdcl1* and arrested in *ΔFgdcl1/2* (**Figure 2**), and the common up-regulated DEGs of *ΔFgdcl1* and *ΔFgdcl1/2* paly more important roles in ascospore discharge than those common down-regulated DEGs (**Figure 3**). To explore whether FgmilRNAs negatively regulate their targets and have a relationship with the physiological changes of *ΔFgdcl1* and *ΔFgdcl1/2*, the novel perithecium-specific milRNAs, which TPM values higher than 10, were selected to predict their targets by psRobot and TargetFinder. After duplicates were removed, 117 putative target genes were identified with the target penalty scores no more than 4 (**Supplementary Table S7**). Statistical results of GO classification and KEGG pathway revealed that no significant GO categories or KEGG pathways were enriched (**Supplementary Figures S7, S8**). Nevertheless, certain KEGG pathways attracted our attention because of their vital roles in sexual reproduction. For example, the peroxisome category (pathway ID: ko04146) is involved in the formation of fruiting bodies and the maturation and germination of sexual spores (Peraza-Reyes and Berteaux-Lecellier, 2013). Fructose and mannose metabolism (pathway ID: ko00051) affects ascospore discharge (Trail et al., 2002; **Supplementary Figure S8** and **Supplementary Table S7**).

MicroRNAs directly degrade their target mRNA in plants, and mediate suppressed mRNA translation in plants and animals. Indeed, based on the transcriptomic data, we calculated the expression level of putative target genes in *ΔFgdcl1* and *ΔFgdcl1/2* in compared to those in the wild-type strain and the correlation between putative targets and FgmiRNAs. In total, 54 putative targets was negatively (*r* < 0) correlated with FgmiRNAs, of which 34 was strong negatively correlation (*r* ≤-0.7) (**Supplementary Table S7**). Besides, the expression patterns of several putative target genes were verified by qRT-PCR (**Supplementary Figure S6B**). Interestingly, mtlD (*FGRRES_16556_M*, the putative target gene of *FgmilR-7dps-61, r* = -0.93), which belongs to the fructose and mannose metabolism pathway, was induced in *ΔFgdcl1* and *ΔFgdcl1/2.* The mRNA transcript level of *FGRRES_16556_M* was notably increased in *ΔFgdcl1* and *ΔFgdcl1/2*, compared with the wild-type strain (**Figure 6D**). These results suggested that perithecium-specific milRNA potentially regulate the sexual development of *F. graminearum.*

## Discussion

RNAi has been found in animals, plants and filamentous fungi, and the components of the RNAi pathway are conserved in a wide range of eukaryotic genomes, while the biological function of these components varies with fungal species. In *Trichoderma atroviride, Botrytis cinerea, M. circinelloides, M. oryzae, C. neoformans*, and *Colletotrichum higginsianum*, the components of the RNAi pathway are involved in the regulation of growth or conidiation (Zamore et al., 2000; Kadotani et al., 2004; Nicolas et al., 2007; de Haro et al., 2009; Janbon et al., 2010; Carreras-Villasenor et al., 2013; Weiberg et al., 2013; Campo et al., 2016). Nevertheless, no biological function has been reported in *Aspergillus nidulans, Saccharomyces castellii*, and *Cryphonectria parasitica* (Segers et al., 2007; Hammond et al., 2008; Drinnenberg et al., 2009). *F. graminearum* also has a functional RNA silencing pathway and contains five RdRP, two Argonaute, and two Dicer genes in its genome (Chen et al., 2015). Previous studies have shown that *Fgdcl1* and *Fgago2* were induced during the sporulation stage, while the deletion of these genes had no effect on the phenotype of *F. graminearum* during the asexual phase (Chen et al., 2015). In the present study, *Fgdcl1* was expressed at a high level in perithecia, and the ascospore discharge of *ΔFgdcl1* was less than that of the wild type strain (**Figures 1, 2**), similar to *N. crassa dcl-1*, which also plays an important role during sexual development (Alexander et al., 2008). Additionally, conidia germination of *ΔFgdcl1* was mildly delayed at the early stage (**Supplementary Figure S2C**). In addition, *Fgago2* is also essential for ascospore discharge (Kim et al., 2015). A previous study showed that perithecia induction inevitably induced asexual sporulation, which may contribute to the high expression of *Fgdcl1* and *Fgago2* during sporulation (Chen et al., 2015; Son et al., 2016). Taken together, these findings show that the ascospore discharge of *ΔFgdcl1* was decreased, and it was blocked in *ΔFgdcl1/2*, suggesting that *Fgdcl1* partly cooperates with *Fgdcl2*.

To explore the mechanism of the inhibitory ascospore discharge of *Fgdcl*, the RNA-sequencing of *ΔFgdcl1* and *ΔFgdcl1/2* revealed common up-regulated DEGs enriched in membrane and transmembrane transport (**Figure 3C** and **Supplementary Figure S5**). The discharge-deficient mutant *Fgamd1*, similar to *ΔFgdcl1/2*, which showed no significant changes in growth and conidiation, also has more than two-thirds up-regulated DEGs related to membrane and transporter activity (Cao et al., 2017). In *F. graminearum*, the turgor pressure of asci is necessary for ascospore discharge, and ion fluxes and sugar alcohol accumulation are related to the generation of turgor pressure, especially in K^+^, Na^+^, Cl^-^ and Ca^++^ ion channels (Trail et al., 2002, 2005; Min et al., 2010). Indeed, this process may contribute to the up-regulated DEGs in sodium ion transmembrane transporter activity, solute: cation symporter activity, and the decrease in glycogen accumulation by glycogen staining (**Figure 3C** and **Supplementary Figures S3A, S5**; da R Campos and Costa, 2010).

Small non-coding RNAs (sRNAs), which range from 17 to 29 nt, act as the core factors mediating the RNAi pathway and are widely identified in eukaryotic organisms (Hamilton and Baulcombe, 1999; Hammond et al., 2000; Zamore et al., 2000). According to extrinsic origins and biological pathways, sRNAs can be grouped into three major categories: miRNA, siRNA and piwi-interacting RNA (piRNA). In addition to these three major small RNAs, new sRNA, such as tasiRNA, natsiRNA, ex-siRNA, endo-siRNA, have also been discovered, and these sRNAs differ in their biogenesis and function. In *F. graminearum*, milRNA and ex-siRNA have been detected in the asexual and sexual stages, respectively (Chen et al., 2015; Son et al., 2017). In asexual stage, 49 milRNAs were detected and 64% loci distributed on intergenic regions (Chen et al., 2015). In the present study, 143 FgmilRNAs were enriched in sexual phases with a strong preference for uracil in the first position and having most of their loci distributed on intergenic regions (54%) (**Figure 5**). Unlike perithecium-specific milRNAs, the asexual stage milRNAs started with 5′ G, and the percentage of exon loci (26%) was decreased compared to that in the sexual stage (44%) (Chen et al., 2015). This finding suggested that the difference between asexual and sexual sRNAs. Moreover, the expression of 4 asexual stage milRNAs have no change in Δ*Fgdcl2*, implying the potential role of *Fgdcl1* in asexual stage. In sexual development, ex-siRNAs, another class of sRNA, also have been reported in *F. graminearum* (Son et al., 2017). Comparing to milRNAs, ex-siRNAs were exonic-specific. Interestingly, certain perithecium-specific milRNAs and ex-siRNAs shared common gene loci and started with 5′ U, implying that the perithecium-specific milRNAs and ex-siRNAs may be similar in biogenesis (**Supplementary Table S6**). MSUD, a silencing mechanism of meiosis, has also been demonstrated in *F. graminearum*, and *Fgsad-1* was required (Son et al., 2011). However, due to the homothallic mating type of *F. graminearum*, less invasive DNA were produced and MSUD-associated siRNAs (masiRNAs) have been reported less often. Indeed, masiRNAs were dispensable for the regulation of sexual development. To the best of our knowledge, *Fgdcl1* and *Fgago2* processed the ex-siRNAs, while *Fgdcl2* and *Fgago1* processed the milRNAs at the asexual stage. In the present study, 19 (13.7%) perithecium-specific milRNAs were detected in *ΔFgdcl1* but with lower expression than in the wild-type, and most of perithecium-specific milRNAs were missing in *ΔFgdcl1/2* (**Figure 6C**). Hence, we suspected that *Fgdcl1* may cooperate with *Fgdcl2* in the biogenesis of perithecium-specific milRNAs. In *N. crassa*, diverse pathways for small RNA biogenesis were discovered, and other factors also play important roles in these pathways, such as RNase III domain-containing protein MRPL3, the exonuclease QIP, and RNA exosome (Lee et al., 2010; Xue et al., 2012). Taken together, these finding make a reasonable case that in addition to pathways dependent to *Fgdcl2* and *Fgago1*, and *Fgdcl1* and *Fgago2*, more pathways for small RNA biogenesis exist, and additional components are involved in these process. A similar relationship of the two dicer genes in perithecium-specific milRNAs biogenesis and sexual development revealed that the milRNAs may play a role in sexual development. MilRNAs negatively regulated gene targeting by seed-region matching and near-perfect matching in animals and plants, respectively (Rhoades et al., 2002; Lewis et al., 2003). In the present study, the bioinformatic prediction of milRNA targets showed that certain target genes related to sexual development and the ascospore discharge pathway. In fungi, the research on target recognization of milRNAs has been less reported. Indeed, the transcription levels of putative targets were identified using qRT-PCR, The results indicated that the putative target genes were increased in *ΔFgdcl1* and *ΔFgdcl1/2*, whilst the expression of milRNAs were decreased or absent (**Figure 6D, Supplementary Figure S6B**, and **Supplementary Table S7**). Hence, this condition may be the reason why more up-regulated DEGs were obtained and may function in ascospore discharge in *ΔFgdcl1* and *ΔFgdcl1/2*, especially in *ΔFgdcl1/2*. The regulation of the ex-siRNA by degrading mRNA transcripts may also be a factor (Son et al., 2017). In addition, no milRNA target genes were found in the ex-siRNA gene loci, indicating that ex-siRNAs have no effect on milRNA targets. Taken together, our results suggested that perithecium-specific milRNAs play important roles in the regulation of sexual development in *F. graminearum*, and the mechanism needs to be further studied.

## Author Contributions

WZ and JC designed the research and wrote the paper. WZ, JW, YW, and JL executed the experiments. WZ, YF, JX, DJ, TC, and JC performed the data and bioinformatic analyses. All authors read and approved the final manuscript.

## Conflict of Interest Statement

The authors declare that the research was conducted in the absence of any commercial or financial relationships that could be construed as a potential conflict of interest. The reviewer ZM and handling Editor declared their shared affiliation.

## Supplementary Material

FIGURE S1 Verification of deletion mutants using Southern blotting and PCR. **(A)** Strategic map of gene disruption construction and restriction map. WT, *Fusarium graminearum* wild-type strain PH-1; gen^r^, geneticin resistance gene cassette; hyg^r^, hygromycin B resistance gene cassette; X, XbaI; P1: the fragment P1, used to identify deletion mutant; P2: the fragment P2, used to identify deletion mutant; P3: the fragment P3, used to identify deletion mutant. The blue line indicates the region of PCR amplification with primers. **(B)** PCR amplification. P1, P2, P3, indicate the fragment P1, P2, P3, respectively. M, marker; the sizes from top to bottom: 5 kb, 3 kb, 2 kb, 1.5 kb, 1 kb, 750 bp, 500 bp, 250 bp, and 100 bp. **(C)** Southern blotting analysis. Genomic DNA from all strains was digested by XbaI. The probe 1 (from hygromycin B resistance gene) and probe 2 (from geneticin resistance gene) labeled with alkaline phosphatase according to the manual. The sizes of the marker are indicated on the left of the blot.

FIGURE S2 The assays of growth, conidiation, conidial germination, and virulence of *F. graminearum* wild-type strain PH-1 and *Fgdcl* deletion mutants. **(A)** Growth rate of *F. graminearum* wild-type strain PH-1 and *Fgdcl* deletion mutants. The mycelial growth rate based on the colony diameter of cultures incubated at 25°C for 3 days. PDA, potato dextrose agar; MM, minimal medium; CM, complete medium. Mean and standard deviation were calculated from three independent replicates, and analyzed with Fisher’s least significant difference (LSD) test. **(B)** Statistics of conidiation of *F. graminearum* wild-type strain PH-1 and *Fgdcl* deletion mutants. The conidial production in carboxymethyl cellulose (CMC) liquid culture at 25°C on a rotary shaker (200 rpm) for 5 days determined with a hemocytometer. Mean and standard deviation were calculated from three independent replicates, and analyzed with Fisher’s LSD test. **(C)** Comparison of conidial germination among *F. graminearum* wild-type strain PH-1 and *Fgdcl* deletion mutants in YEPD at 25°C for 4 and 6 h. hpi, hour post-inoculation. The lines indicate the mean value. The mean values were calculated from five independent replicates. **(D)** Flowering wheat heads were inoculated with the conidial suspension of *F. graminearum* wild-type strain PH-1 and *Fgdcl* deletion mutants. The infected wheat heads were imaged after 21 days of inoculation. **(E)** Corn silks were inoculated with the mycelial plugs of *F. graminearum* wild-type strain PH-1 and *Fgdcl* deletion mutants. Images were captured after 6 days of inoculation.

FIGURE S3 Comparison of colony morphology, conidia morphology, perithecia, asci, and the stress procession of *F. graminearum* wild-type strain PH-1 and *Fgdcl* deletion mutants. **(A)** Comparison of the colony morphology, conidia, perithecia, asci. The colony morphology was imaged after 3 days of incubation on PDA at 25°C. One milliliter of a conidia suspension of each strain was observed after incubation in CMC liquid culture at 25°C on a rotary shaker (200 rpm) for 5 days; scale bar = 10 μm. The images of the morphology of perithecia and asci were captured at 7 dps, and the asci were stained by KI-I_2_; scale bar: 200 μm and 20 μm, respectively. **(B)** The tolerance to osmotic, kalium, calcium, peroxide, and cell wall damaging agents stress among the wild-type strain PH-1 and mutants. PH-1 and mutants were incubated on the PDA plates containing 1 M glucose, 1 M sorbitol, 1 M NaCl, 1 M KCl, 0.5 M CaCl_2_, 0.02% SDS, 0.05% H_2_O_2_, and 0.2 mg/ml Congo red. The colony morphology of PDA plates and PDA containing 1 M glucose, 1 M sorbitol, 1 M NaCl, and 1 M KCl were imaged after 2 days, the others were imaged after 3 days.

FIGURE S4 Comparison of sexual development among the wild-type strain PH-1, *ΔFgdcl1-1, ΔFgdcl1-2, ΔFgdcl1/2-1, and ΔFgdcl1/2-2.* **(A)** Comparsion of the cirrhi and ascospore discharge of wild-type strain PH-1, *ΔFgdcl1-1, ΔFgdcl1-2, ΔFgdcl1/2-1*, and *ΔFgdcl1/2-2*. Each strain was inoculated on carrot medium, and the cirrhi were captured at 12 days post self-crossing, Scale bar = 500 μm. Ascospore discharge was assayed with 7-day-old perithecia. The images were captured 14 h after ascospores had been released. **(B)** Statistical analysis of asci rosettes of 7-day-old perithecia. Ascus morphology was conducted after 7 days post self-crossing. A total of 20 asci rosettes were observed and classified in each strain with light microscopy. Line bars indicate standard errors from three repeated experiments.

FIGURE S5 The network of enriched Gene ontology (GO) terms of common up-regulated DEGs between *ΔFgdcl1* and *ΔFgdcl1/2* according to REVIGO. Bubble color and size indicate the adjusted *p*-value and the frequency of the GO term in the underlying GO database, respectively. Highly similar GO terms are linked by edges in the graph, where the line width indicates the degree of similarity.

FIGURE S6 Verification of the transcript levels of enriched GO terms genes **(A)** and the putative target genes of microRNA-like RNAs (milRNAs) **(B)** using qRT-PCR. The black bars reflect the results of qRT-PCR, and the gray lines indicate the data of transcriptome sequencing. *FGRRES_17029_M, FGRRES_00220, FGRRES_04240*, and *FGRRES_02426* belong to transmembrane transporter; *FGRRES_11032* and *FGRRES_03569* involve in galactose oxidase activity; *FGRRES_06554* and *FGRRES_06942* are peroxisome (k04146); *FGRRES_09366, FGRRES_16556_M* class into starch and sucrose metabolism (k00500) and fructose and mannose metabolism (k00051), respectively.

FIGURE S7 Gene ontology classification of target genes. No GO term was significantly enriched due to the adjusted *p*-values greater than 0.05. BP, biological process; CC, cellular component; MF, molecular function.

FIGURE S8 KEGG classification of target genes. The bracketed number indicates the target number in these KEGG pathways. No KEGG pathways were significantly enriched due to adjusted *p*-values greater than 0.05.

TABLE S1 PCR primers used in this study.

TABLE S2 Statistics of the clean reads and the mapped reads in transcriptome of the wild-type strain PH-1, *ΔFgdcl1*, and *ΔFgdcl1/2*.

TABLE S3 The differentially expressed genes (DEGs) in *ΔFgdcl1* and *ΔFgdcl1/2.*

TABLE S4 Summary statistics of the clean reads and the mapped reads in small RNA libraries of the wild-type strain PH-1, *ΔFgdcl1*, and *ΔFgdcl1/2*.

TABLE S5 The annotations of mapped small reads of the wild-type strain, *ΔFgdcl1* and *ΔFgdcl1/2.*

TABLE S6 The prediction and expression of novel perithecium-specific microRNA-like RNA (milRNAs).

TABLE S7 The prediction, expression, and annotation of target genes.
