# Supplementary figures and images for: Dicer-Like Proteins Regulate Sexual Development via the Biogenesis of Perithecium-Specific MicroRNAs in a Plant Pathogenic Fungus Fusarium graminearum
Source: Front Microbiol. 2018 Apr 26;9:818. doi: 10.3389/fmicb.2018.00818 (PMC5932338; doi:10.3389/fmicb.2018.00818)

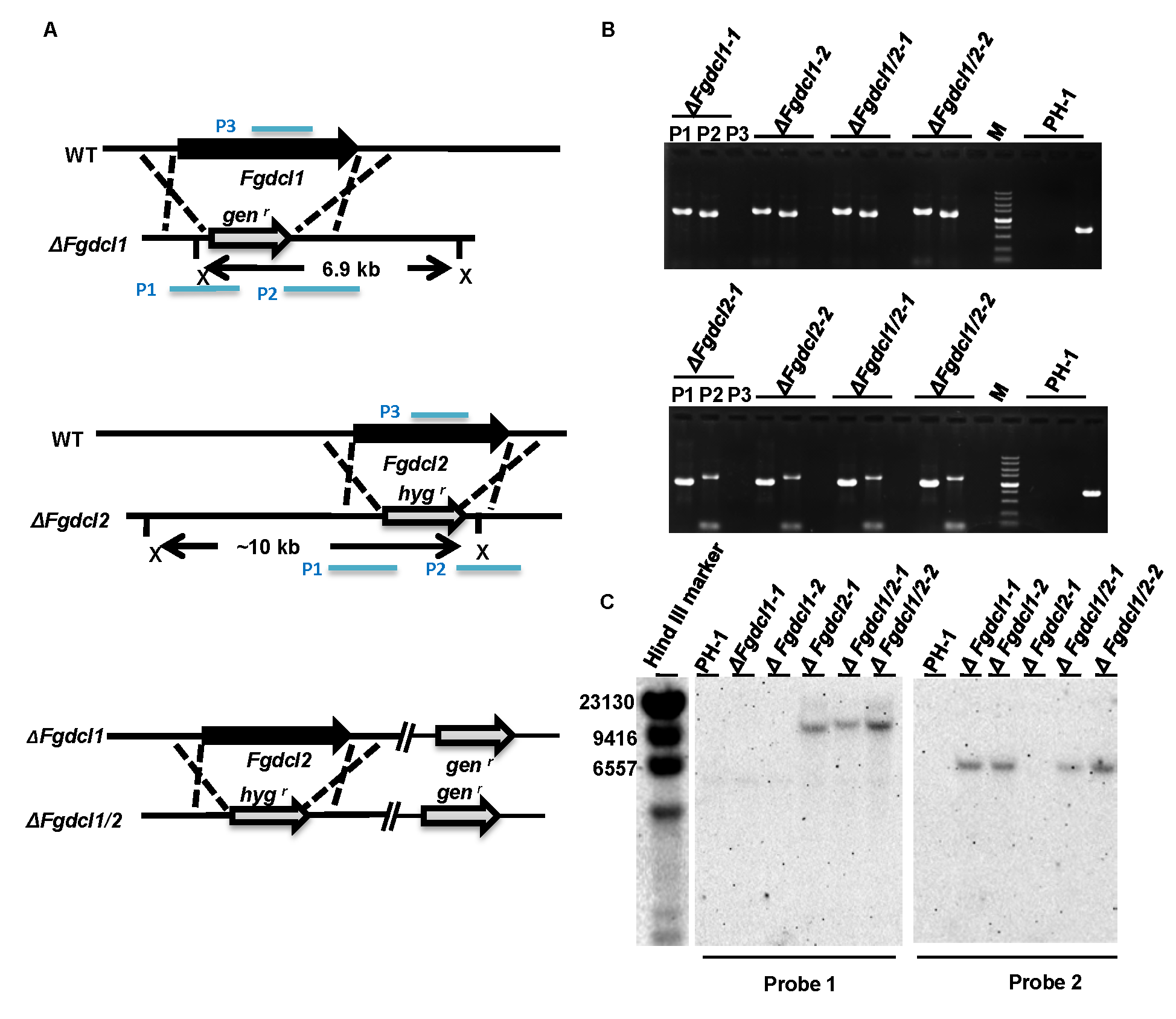

Supplement: FIGURE S1 — Verification of deletion mutants using Southern blotting and PCR. (A) Strategic map of gene disruption construction and restriction map. WT, Fusarium graminearum wild-type strain PH-1; genr, geneticin resistance gene cassette; hygr, hygromycin B resistance gene cassette; X, XbaI; P1: the fragment P1, used to identify deletion mutant; P2: the fragment P2, used to identify deletion mutant; P3: the fragment P3, used to identify deletion mutant. The blue line indicates the region of PCR amplification with primers. (B) PCR amplification. P1, P2, P3, indicate the fragment P1, P2, P3, respectively. M, marker; the sizes from top to bottom: 5 kb, 3 kb, 2 kb, 1.5 kb, 1 kb, 750 bp, 500 bp, 250 bp, and 100 bp. (C) Southern blotting analysis. Genomic DNA from all strains was digested by XbaI. The probe 1 (from hygromycin B resistance gene) and probe 2 (from geneticin resistance gene) labeled with alkaline phosphatase according to the manual. The sizes of the marker are indicated on the left of the blot. [file Image_1.tif]

Figure S2

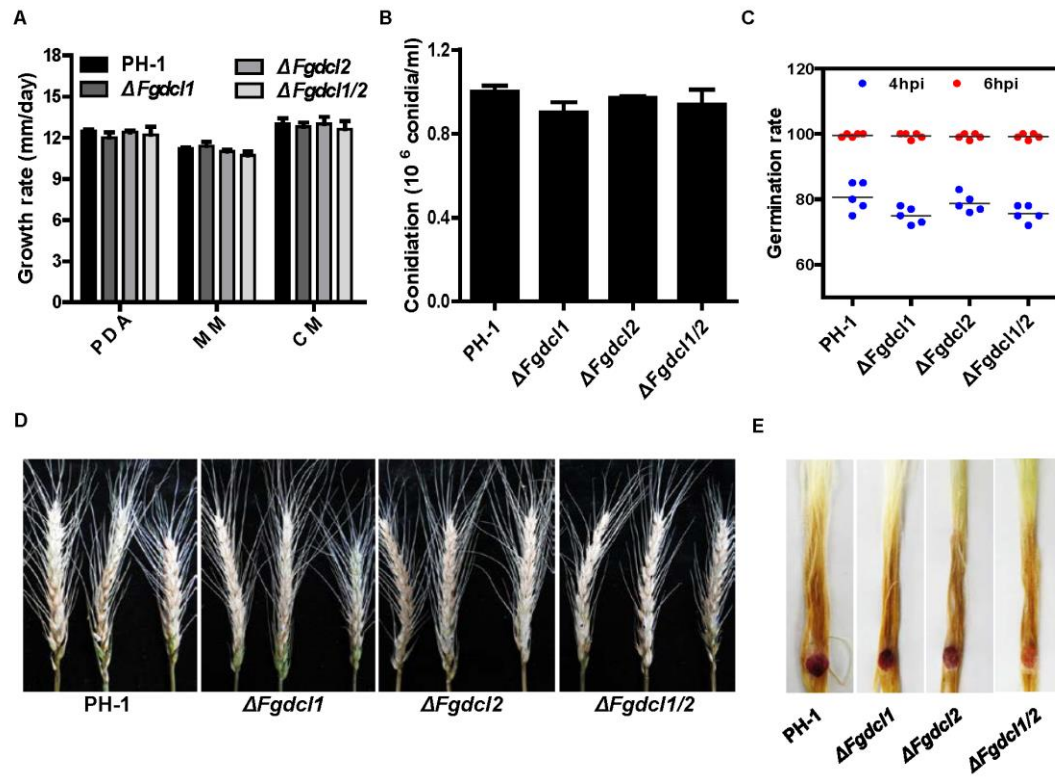

Figure S3

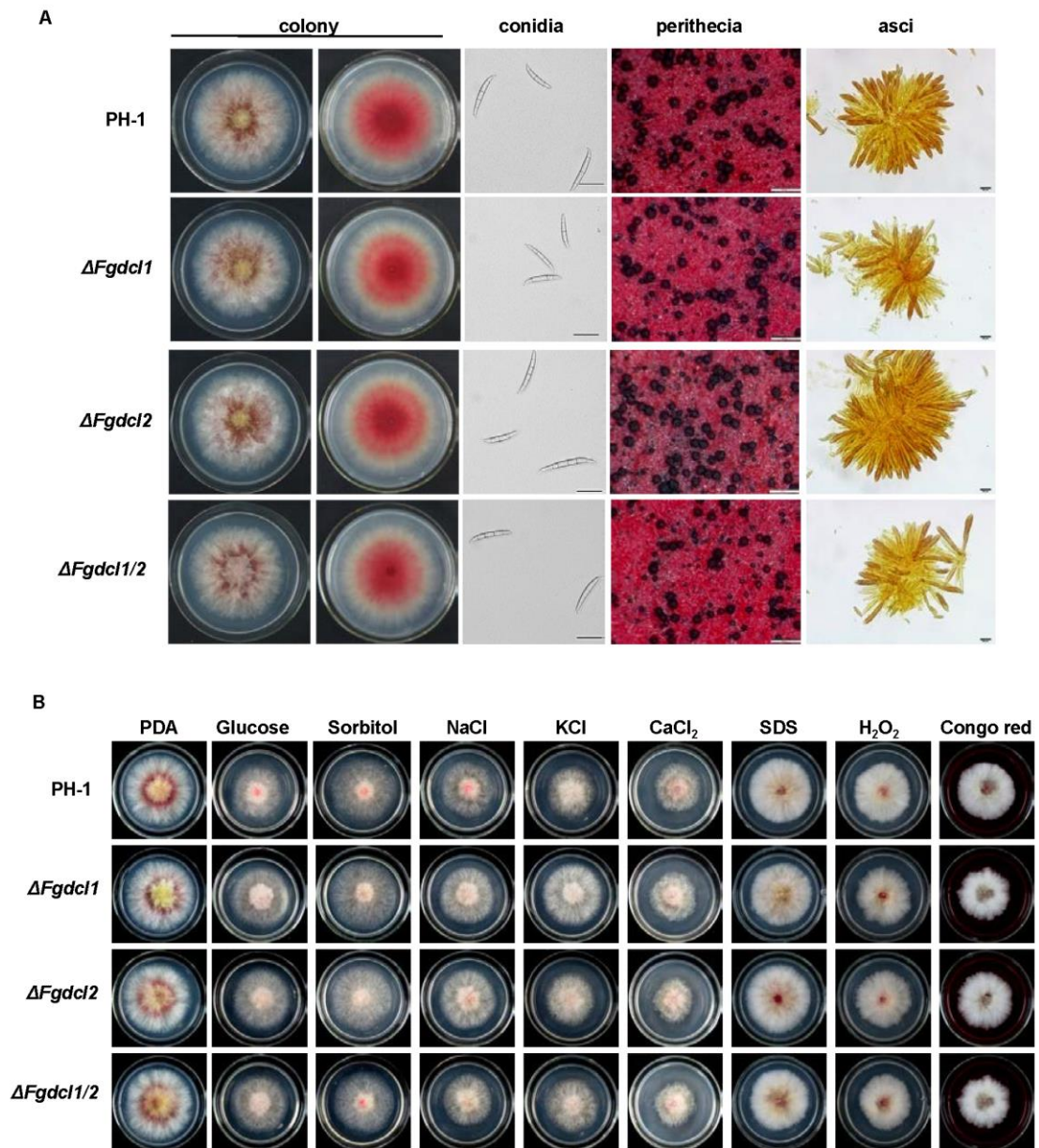

Figure S4

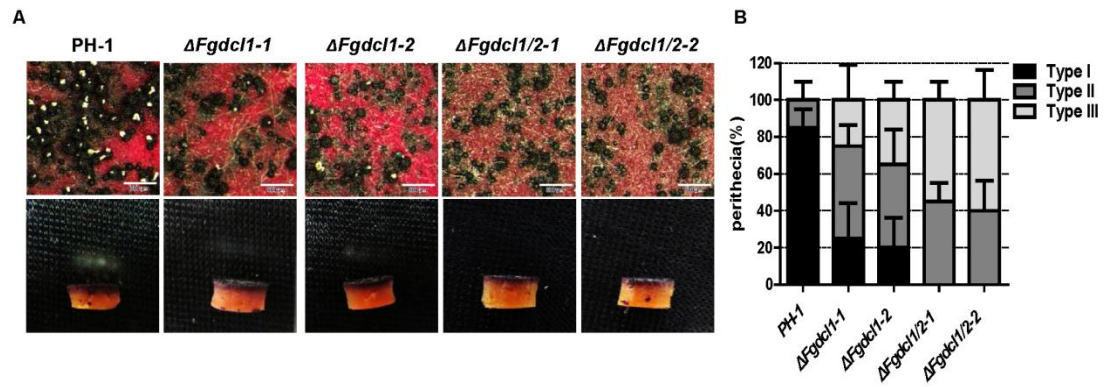

**Figure S5**

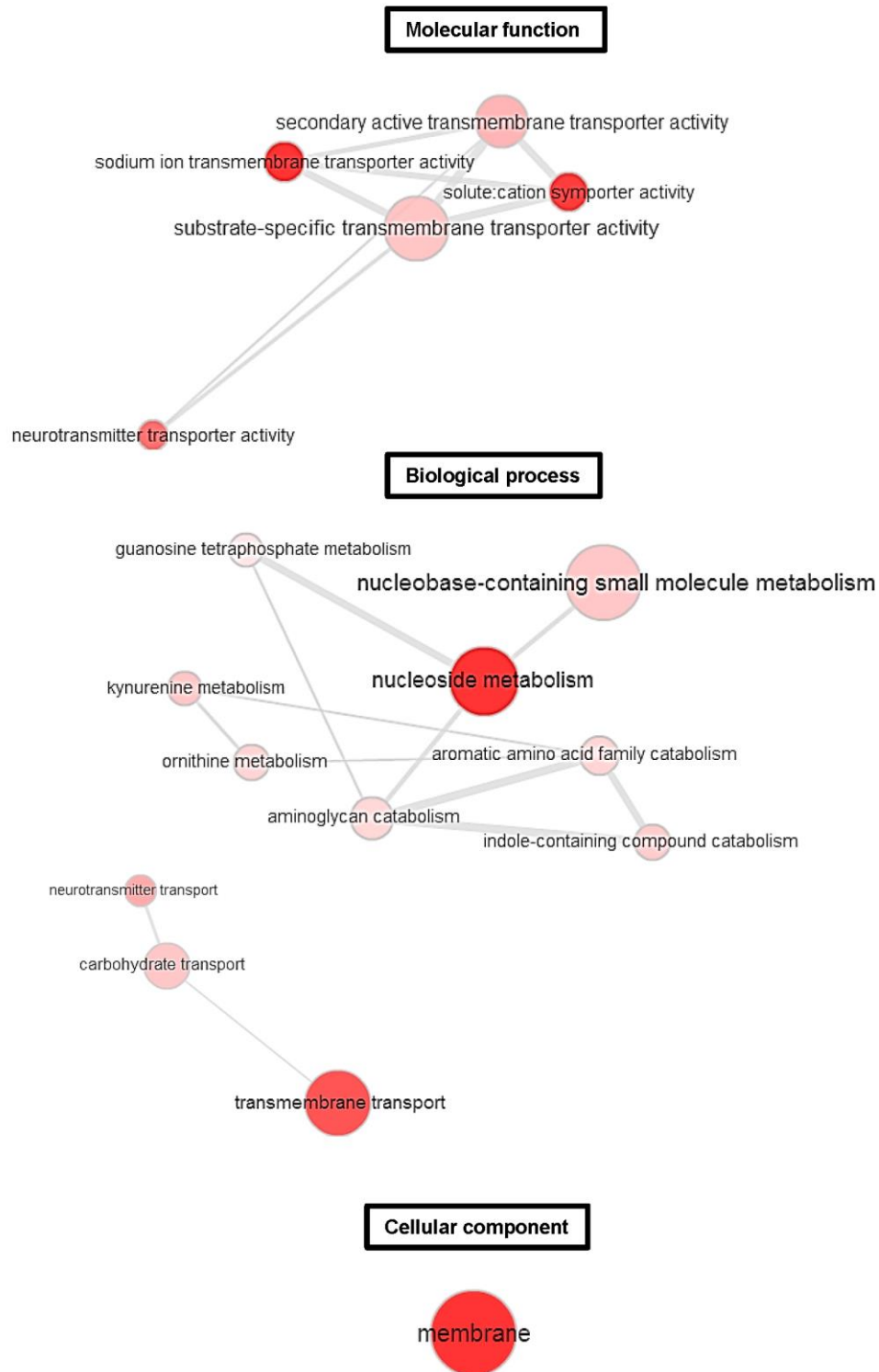

Figure S6

A

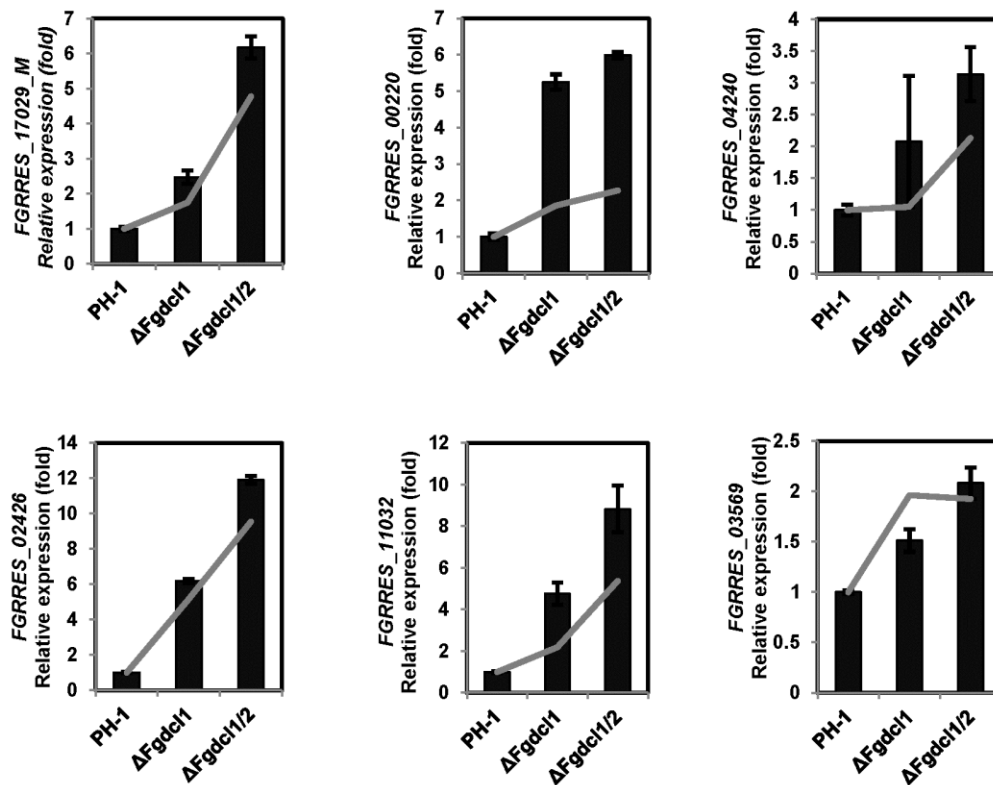

B

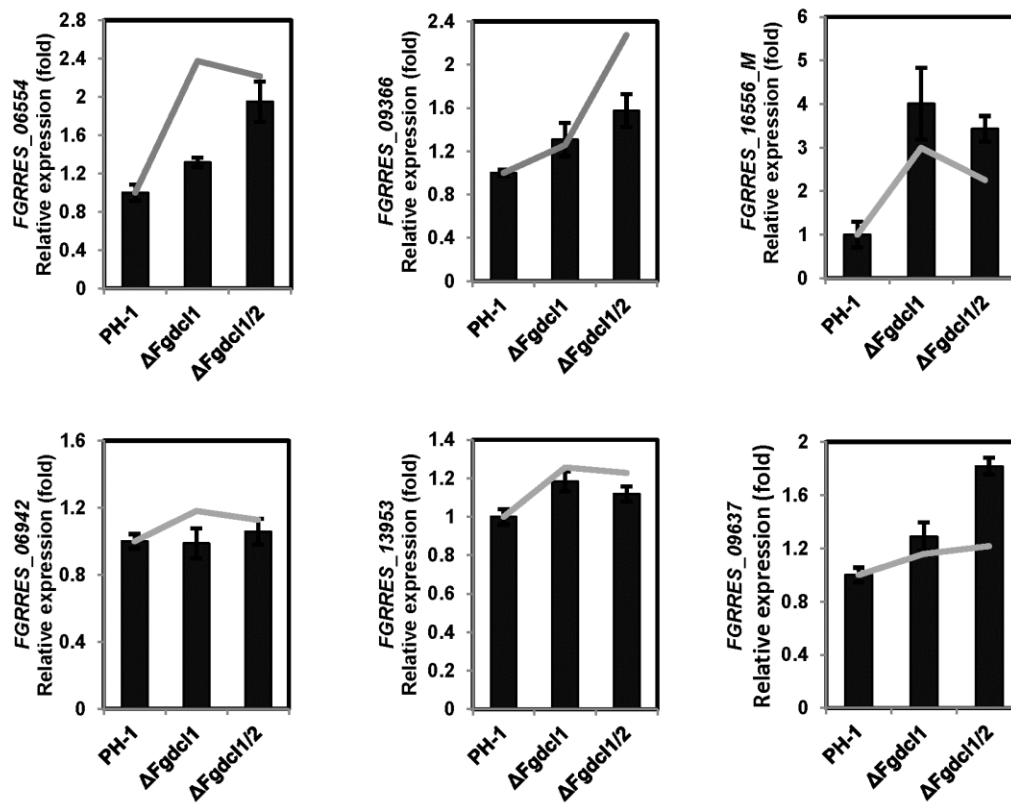

**Figure S7**

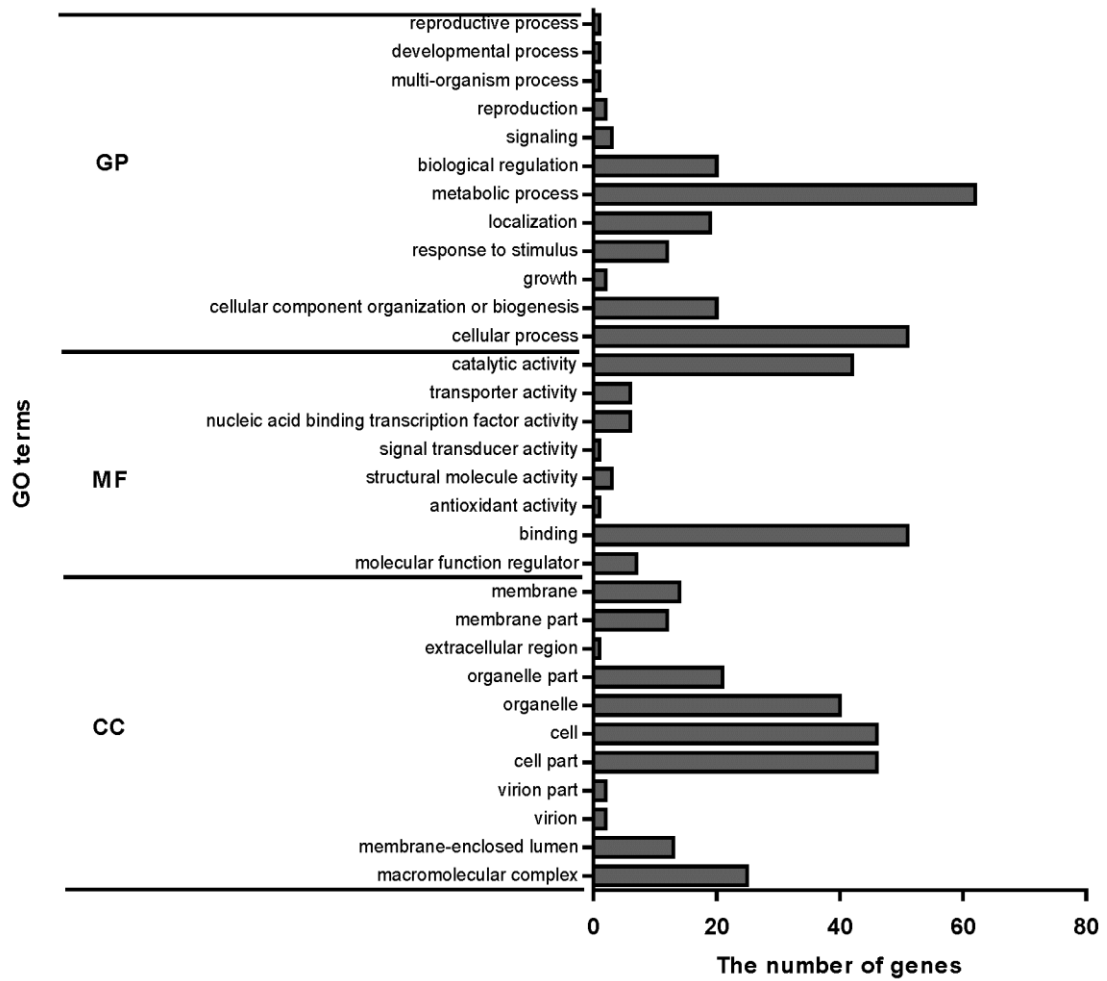

**Figure S8**

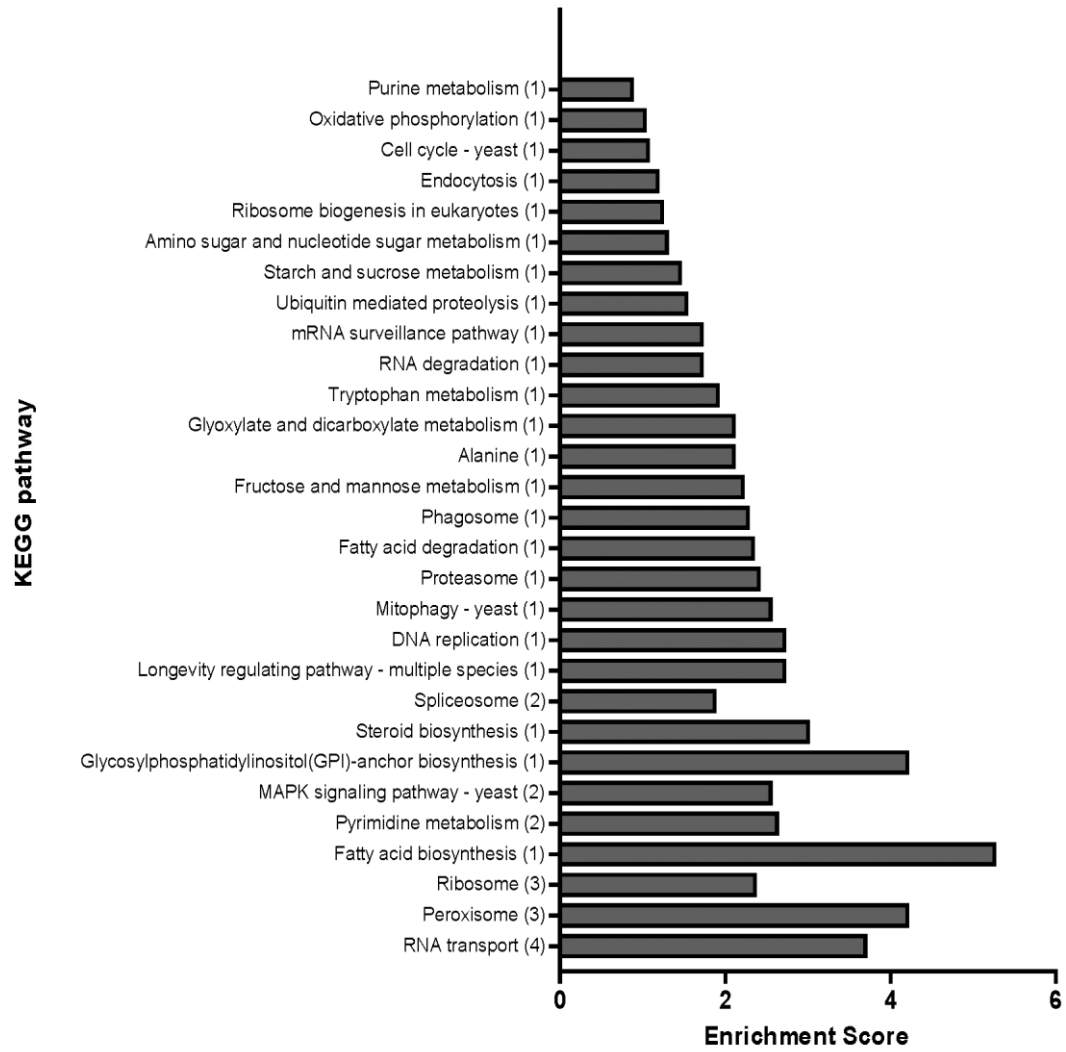

Supplement: FIGURE S2 — The assays of growth, conidiation, conidial germination, and virulence of F. graminearum wild-type strain PH-1 and Fgdcl deletion mutants. (A) Growth rate of F. graminearum wild-type strain PH-1 and Fgdcl deletion mutants. The mycelial growth rate based on the colony diameter of cultures incubated at 25°C for 3 days. PDA, potato dextrose agar; MM, minimal medium; CM, complete medium. Mean and standard deviation were calculated from three independent replicates, and analyzed with Fisher’s least significant difference (LSD) test. (B) Statistics of conidiation of F. graminearum wild-type strain PH-1 and Fgdcl deletion mutants. The conidial production in carboxymethyl cellulose (CMC) liquid culture at 25°C on a rotary shaker (200 rpm) for 5 days determined with a hemocytometer. Mean and standard deviation were calculated from three independent replicates, and analyzed with Fisher’s LSD test. (C) Comparison of conidial germination among F. graminearum wild-type strain PH-1 and Fgdcl deletion mutants in YEPD at 25°C for 4 and 6 h. hpi, hour post-inoculation. The lines indicate the mean value. The mean values were calculated from five independent replicates. (D) Flowering wheat heads were inoculated with the conidial suspension of F. graminearum wild-type strain PH-1 and Fgdcl deletion mutants. The infected wheat heads were imaged after 21 days of inoculation. (E) Corn silks were inoculated with the mycelial plugs of F. graminearum wild-type strain PH-1 and Fgdcl deletion mutants. Images were captured after 6 days of inoculation. [file Data_Sheet_1.pdf]
